# From Cytology to Frozen Section: Diagnostic Challenges in Riedel’s Thyroiditis—A Case Report and Brief Literature Review

**DOI:** 10.3390/jcm15072529

**Published:** 2026-03-26

**Authors:** Diana-Raluca Streinu, Andreea Bena, Ion Icma, Ivan Codrut, Călin Muntean, Iuliana-Anamaria Trăilă, Dana Liana Stoian

**Affiliations:** 1 Department of Doctoral Studies, Victor Babes University of Medicine and Pharmacy, 300041 Timisoara, Romania; 2 Discipline of Endocrinology, Victor Babes University of Medicine and Pharmacy, 300041 Timisoara, Romania; 3 First Surgery Clinic, “Pius Brinzeu” Clinical Emergency Hospital, 300723 Timisoara, Romania; 4 Abdominal Surgery and Phlebology Research Center, Victor Babes University of Medicine and Pharmacy, 300041 Timisoara, Romania; 5 Department X of Surgery, Victor Babes University of Medicine and Pharmacy Timisoara, 300041 Timisoara, Romania; 6 Medical Informatics and Biostatistics, Victor Babes University of Medicine and Pharmacy, 300041 Timisoara, Romania; 7 Department of Microscopic Morphology Genetics Discipline, Center of Genomic Medicine, Victor Babes University of Medicine and Pharmacy Timisoara, 300041 Timisoara, Romania; 8 Dr. D Medical Center, Center for Advanced Ultrasound Evaluation, 300029 Timisoara, Romania

**Keywords:** Riedel’s thyroiditis, case report, thyroidectomy, frozen section, FNAC

## Abstract

**Background**: Riedel’s thyroiditis is a rare fibrosing thyroid disorder that remains one of the most difficult to diagnose, often being initially interpreted as malignant due to its clinical, radiological, and histopathological similarities with anaplastic carcinoma or other infiltrative thyroid diseases. Preoperative investigations, including fine-needle aspiration cytology (FNAC), may be misleading and contribute to an erroneous diagnosis of cancer. **Methods**: We report the case of a 44-year-old woman presenting with a rapidly enlarging, hard goiter associated with compressive symptoms and cytological findings suspicious for papillary thyroid carcinoma (Bethesda V). Based on these findings and the multidisciplinary team’s assessment, surgical intervention was undertaken. Intraoperatively, the thyroid gland was densely fibrotic and adherent to adjacent structures, prompting frozen-section analysis that revealed a benign fibroinflammatory process consistent with Riedel’s thyroiditis. This intraoperative finding guided the surgical team toward a near-total thyroidectomy, preventing unnecessary radical excision. **Results:** The paraffin-embedded section confirmed the diagnosis. Postoperative recovery was favorable, with complete resolution of compressive symptoms. **Conclusions**: This case highlights the persistent diagnostic challenges of Riedel’s thyroiditis and illustrates how intraoperative frozen-section examination can contribute to differentiating it from malignancy when preoperative findings remain inconclusive. A multidisciplinary approach and surgical expertise are essential in tailoring the extent of resection, preventing complications, and achieving both diagnostic confirmation and symptom relief.

## 1. Introduction

Riedel’s thyroiditis is a rare chronic inflammatory disease of the thyroid gland, characterized by extensive fibroinflammatory replacement of the normal thyroid parenchyma. The fibrotic process frequently extends beyond the thyroid capsule, infiltrating adjacent neck structures and, in advanced cases, leading to airway compression or obstructive symptoms [1]. It has an estimated incidence of approximately 1 in 100,000 individuals, occurring more frequently in women between 30 and 50 years of age [2,3]. While environmental and lifestyle factors have been proposed to influence thyroid disorders, the etiology of Riedel’s thyroiditis remains poorly understood [4,5,6,7]. The condition is, however, strongly associated with autoimmune and fibroinflammatory processes and, in a subset of cases, with IgG4-related systemic disease [8,9].

The main diagnostic challenge lies in differentiating Riedel’s thyroiditis from anaplastic thyroid carcinoma, as both entities share overlapping clinical, radiologic, and pathological features, including a hard, fixed thyroid mass and local invasion. Fine-needle aspiration cytology (FNAC) is often inconclusive and may be misleading, since the extensive fibrosis and paucity of follicular cells can mimic malignancy or other chronic thyroiditis subtypes [10,11].

Furthermore, not all cases of Riedel’s thyroiditis show elevated serum IgG4 or increased IgG4-positive plasma cell infiltration. Cases with normal IgG4 levels can be challenging to distinguish from malignant thyroid disease both clinically and cytologically [3]. This underscores the heterogeneity of Riedel’s thyroiditis and highlights the importance of correlating histopathological findings—particularly intraoperative frozen section results—with the clinical and imaging context.

This article describes a rare case of Riedel’s thyroiditis in a 44-year-old female patient, illustrating the challenges in establishing a correct preoperative diagnosis and highlighting how intraoperative frozen-section examination may assist in guiding the extent of surgical management.

## 2. Case Report

### 2.1. Clinical Presentation and Laboratory Evaluation

A 44-year-old female patient was referred to the Surgery Department after prior endocrinological evaluation with an initial presumptive diagnosis of Riedel’s thyroiditis. However, the rapidly enlarging goiter, invasive clinical features, and cytology findings had raised concern for a possible malignant thyroid process, including anaplastic thyroid carcinoma. On admission, she presented with a medium-sized goiter, fixed to adjacent cervical structures and associated with intermittent dyspnea, dysphonia, and dysphagia for solid foods, that had progressively worsened. The patient also had a known diagnosis of pseudomyxoma of the left vocal cord.

Her medical history was notable for type 1 diabetes mellitus, distal sensory neuropathy, chronic kidney disease, and hepatic steatosis. She had been receiving levothyroxine 150 μg daily since 2014.

Physical examination revealed a eutrophic female with stable vital signs. Palpation identified a firm, fixed, anterior cervical mass measuring approximately 7 × 5 cm, with the left lobe measuring 3 × 5 cm and the right lobe 4 × 5 cm. The mass was non-tender, and carotid pulsations were palpable bilaterally.

Laboratory evaluation showed elevated thyroid-stimulating hormone (TSH) of 9.50 mIU/L (reference: 0.46–4.68) and normal free thyroxine (FT4) of 24.2 pmol/L (reference: 10.0–28.2). Thyroid antibodies were markedly elevated, with thyroglobulin antibodies (anti-TG) at 1885 IU/mL (reference: 0–115) and thyroid peroxidase antibodies (anti-TPO) exceeding 1000 IU/mL (reference: 0–34).

Complete blood count showed a mild leukocytosis (white blood cells: 10,090/µL; reference: 4000–9500) and normocytic anemia (hemoglobin: 10.5 g/dL; reference: 11.5–15), with normal eosinophil and platelet counts. Serum calcium was slightly reduced at 8.3 mg/dL (reference: 8.4–10.2), and fibrinogen was elevated at 604 mg/dL (reference: 200–393).

Serum IgG4 concentration was within the normal range at 0.271 g/L (reference: 0.030–2.010).

The main laboratory findings are summarized in Table 1.

Prior to referral to the surgical department, the patient had undergone endocrinological evaluation, including thyroid ultrasonography, chest X-ray, abdominal ultrasound and fine-needle aspiration cytology.

### 2.2. Preoperative Ultrasonographic Assessment

A preoperative B-mode ultrasound and shear-wave elastography (SWE) evaluation were performed.

**Figure 1:**
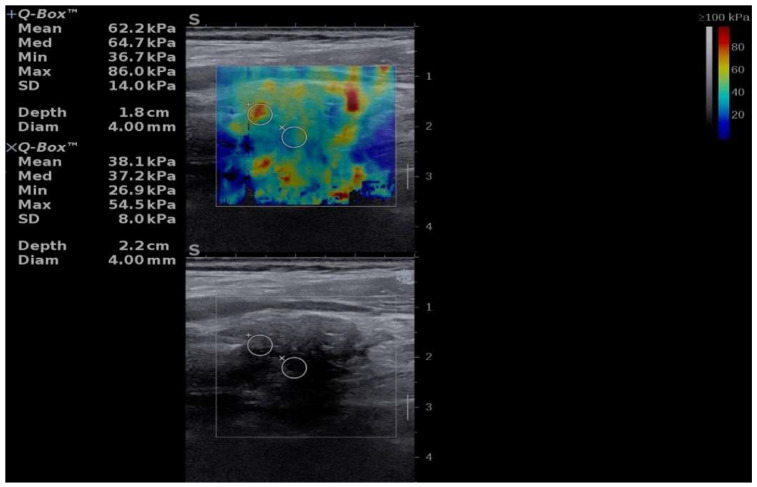
Illustration of B-mode ultrasound (**bottom image**) and two-dimensional Shear-Wave Elastography (**upper image**).

On longitudinal B-mode imaging, the left thyroid lobe appeared markedly hypoechoic with a diffuse, inhomogeneous echotexture, consistent with extensive fibrosis (Figure 1).

Quantitative two-dimensional shear-wave elastography (2D-SWE) revealed a heterogeneous stiffness pattern, with predominantly blue areas indicating softer regions and scattered red zones corresponding to increased stiffness. The elastography measurements were expressed in kilopascals (kPa) and displayed as mean, median, minimum, maximum, and standard deviation values, along with the diameter and depth of the selected region of interest (Figure 1).

A chest X-ray was performed, which revealed no pathological findings, including mediastinal abnormalities or radiographic evidence of intrathoracic thyroid extension.

An abdominal ultrasound was also performed to assess potential systemic involvement and revealed no hepatic, pancreatic, or retroperitoneal fibrosis suggestive of IgG4-related disease.

### 2.3. Fine-Needle Aspiration Cytology (FNAC)

**Figure 2:**
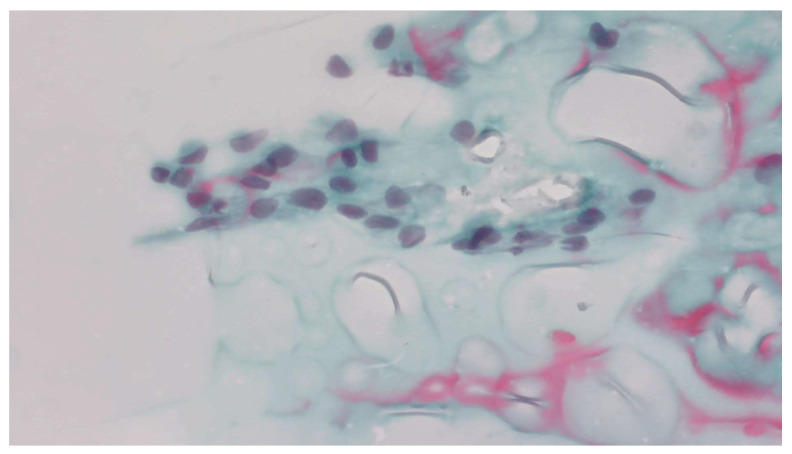
Cytological smear from fine-needle aspiration showing elongated cells with pale, irregular nuclei and overlapping, mimicking papillary carcinoma (magnification—400×).

Preoperative FNAC was performed. Cytological examination revealed a two-dimensional cellular cluster composed of elongated cells with pale, irregular nuclei showing overlap and occasional nuclear grooves (Figure 2). Based on these features, the conventional smear cytology was initially interpreted as Bethesda category V—suspicious for papillary thyroid carcinoma.

In addition to the conventional smear, aspirated material from the same FNAC was processed using a commercial CytoMatrix block (developed by UCS Diagnostics Srl, Rome, Italy and the Campus Bio-Medico University of Rome, Italy) to improve sample adequacy [12]. The CytoMatrix preparation was markedly hypocellular, containing predominantly fibrotic material and lacking representative follicular epithelial elements, and was therefore considered non-diagnostic.

Given the rapidly progressive goiter and severe compressive symptoms, coupled with inconclusive preoperative findings, the differential diagnosis was established between Riedel’s thyroiditis and anaplastic carcinoma, with significant concern for the latter. Although glucocorticoid therapy was initially considered, it was deferred pending cytological clarification. FNAC revealed cytological features interpreted as suspicious for papillary thyroid carcinoma (Bethesda category V). Based on these findings, the rapidly enlarging goiter, the presence of severe compressive symptoms, and the clinical suspicion of malignancy, the multidisciplinary team decided to proceed with surgical intervention.

### 2.4. Surgical Management and Intraoperative Findings

**Figure 3:**
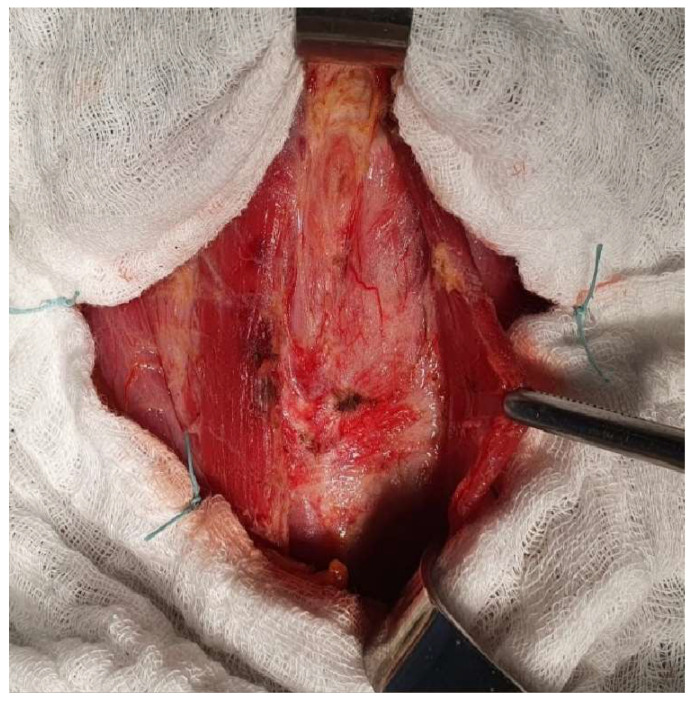
Anterior cervical intraoperative view showing dense fibrosis of the thyroid gland.

A neck collar incision was made, exposing the planes underneath the skin and subcutaneous tissue. The muscular planes dissection was difficult due to the diffuse infiltration of the strap muscles. A hard and fibrous infiltrated thyroid gland is exposed, fixated to the sternothyroid and omohyoid muscles (shown in Figure 3).

**Figure 4:**
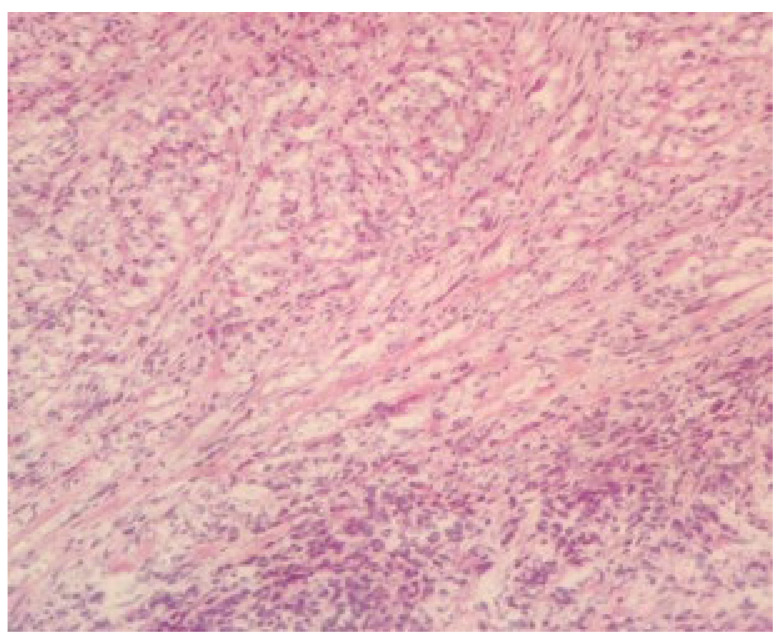
Frozen section of the surgical specimen (magnification—200×).

Dissection proved technically challenging due to the diffuse infiltration and fibrosis extending into the strap muscles and surrounding soft tissues, creating an intraoperative impression of an infiltrative process. These findings were atypical and raised diagnostic uncertainty—although anaplastic carcinoma remained a concern, the extensive fibrosis also revived the suspicion of Riedel’s thyroiditis. To clarify the nature of the lesion and guide the extent of resection, an intraoperative frozen section was obtained. Microscopic examination revealed densely fibrotic and collagenous tissue with chronic inflammatory infiltrate containing eosinophils, but no malignant cellular features (Figure 4). The overall morphology favored the diagnosis of Riedel’s thyroiditis, allowing the surgical team to limit dissection and avoid unnecessary extensive surgery, given the risk of complications associated with diffuse fibrosis.

**Figure 5:**
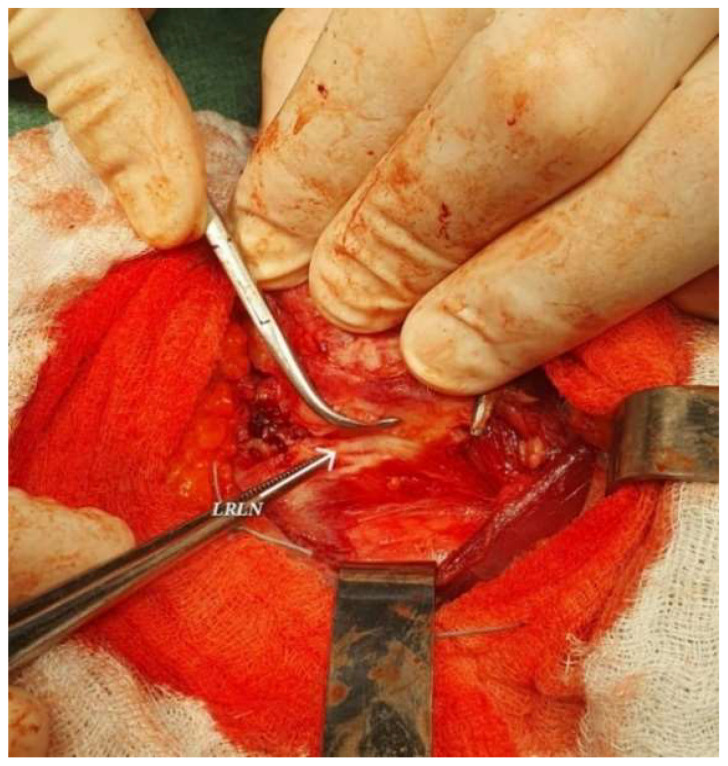
Intraoperative view showcasing the left recurrent laryngeal nerve (LRLN).

A near-total thyroidectomy with excision of the pyramidal lobe was subsequently performed. The procedure was technically demanding due to the extensive and diffuse fibrosis adherent to surrounding vital structures. To minimize the risk of serious complications, a small amount of thyroid tissue was deliberately left in place posteriorly and bilaterally, ensuring preservation of the recurrent laryngeal nerves and adjacent parathyroid glands (shown in Figure 5).

**Figure 6:**
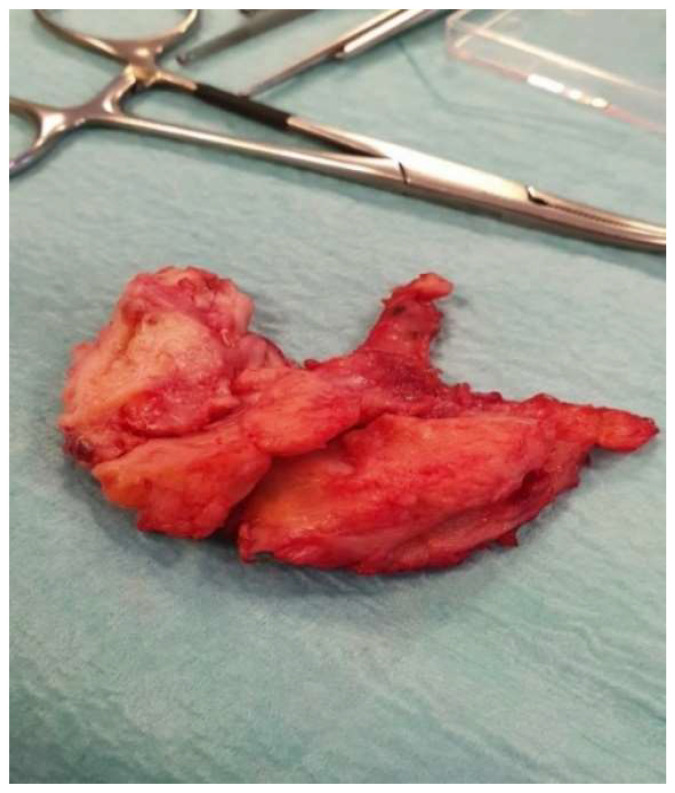
Excised fibrous thyroid gland with pyramidal lobe.

The excised thyroid gland (shown in Figure 6) was sent for histopathological analysis.

A 12 mm silicone drain tube was placed which was removed the next day postoperatively.

Immediately postoperatively, the patient presented with stridor, and orotracheal intubation was mandatory. The following day, the patient was extubated.

The ongoing evolution of the patient was favorable, with the persistency of mild dysphonia (most likely due also to the vocal cord pseudomyxoma), and mild dyspnea, with normal oxygen saturation levels. There was also a small temporary drop in serum calcium levels, with a value of 7.4 mg/dL (reference: 8.4–10.2 mg/dL) on the first postoperative day, which was corrected with intravenous calcium supplementation, followed by a progressive increase to 8.4 mg/dL by the sixth postoperative day.

The final paraffin-embedded examination report concluded a diagnosis of chronic invasive fibrosing thyroiditis (Riedel’s thyroiditis), exhibiting features overlapping with the fibrous variant of chronic autoimmune (lymphocytic) thyroiditis in its atrophic stage. Correlating the histopathological findings with the clinical and intraoperative data confirmed the final diagnosis of Riedel’s thyroiditis.

The patient was referred for an endocrinology consult, during which methylprednisolone 16 mg daily was initiated, along with calcium and vitamin D supplementation, while maintaining the previous levothyroxine dose of 150 µg daily. The patient was discharged on the 8th day postoperatively.

The postoperative evaluation at two weeks after discharge revealed normal serum calcium levels.

The patient’s progress remained favorable, with the resolution of dyspnea and dysphonia observed over the following three months post-surgery. As a result, the decision was made to discontinue the glucocorticoid treatment three months after the procedure. No changes were made to the Levothyroxine dosage over the 18-month period following surgery.

One and a half years later, follow-up showed complete resolution of the compressive symptoms, with no further complications; the patient was fully functional, with continued 150 mcg daily Levothyroxine.

## 3. Discussion

Riedel’s thyroiditis remains one of the most challenging entities to diagnose within thyroid pathology, predominantly affecting women [3,13]. Owing to its rarity and the limited number of reported cases in the literature, the condition continues to pose diagnostic and therapeutic difficulties even for experienced clinicians [2]. In our institution, two retrospective studies conducted across all three surgical departments of the Timisoara Emergency County Hospital analyzed a total of 1484 patients who underwent thyroid surgery between 2018 and 2023, with the present case representing the only documented instance of Riedel’s thyroiditis during that period [14,15]. Similarly, a large-scale review from the Mayo Clinic, encompassing 56,700 thyroidectomy patients between 1921 and 1985, identified only 37 confirmed cases of Riedel’s thyroiditis [2].

Establishing an accurate differential diagnosis between Riedel’s thyroiditis and anaplastic thyroid carcinoma is crucial, as both entities can present with a rapidly enlarging, hard, fixed thyroid mass and local invasion. Although anaplastic carcinoma most frequently occurs in individuals over 60 years of age, it may also appear in younger patients [16,17,18,19]; therefore, age alone cannot be used to exclude malignancy. When additional findings—such as extensive fibrosis without cytologic atypia—are present, they may support a diagnosis of Riedel’s thyroiditis, but rarely provide certainty before surgery.

The etiology of Riedel’s thyroiditis remains uncertain. The most widely accepted hypothesis proposes an autoimmune or systemic fibro-inflammatory origin, as the disease has been associated with Hashimoto’s thyroiditis, Graves’ disease, and other autoimmune disorders [1,9,13]. This concept aligns with the overlap between Riedel’s thyroiditis and IgG4-related disease (IgG4-RD), characterized by dense fibrosis and chronic inflammatory infiltration. However, not all cases demonstrate elevated serum IgG4 or IgG4-positive plasma-cell infiltration, suggesting heterogeneity in pathogenesis [20]. In our patient, the coexistence of type 1 diabetes mellitus supports an autoimmune background, while the normal IgG4 concentration suggests an isolated thyroidal process rather than systemic IgG4-RD.

Despite advances in imaging and cytology, no preoperative investigation can definitively identify Riedel’s thyroiditis. Delays in patient presentation or diagnostic evaluation have also been reported to complicate the assessment of complex pathologies requiring coordinated, multidisciplinary decision-making [21]. Most published reports describe similar diagnostic uncertainty [11,22,23]. In our case, FNAC was performed, but it yielded a misleading result: the smear showed elongated cells with pale, irregular nuclei, initially interpreted as suspicious for papillary carcinoma (Bethesda V). This pitfall has been described in several studies, as intense fibrosis and a scarcity of epithelial elements often lead to overinterpretation of fibroblastic spindle cells as malignant. Consequently, FNAC may not only fail to clarify the diagnosis but also strengthen a false impression of carcinoma, as occurred here [24,25,26,27].

The diagnostic limitations of FNAC were further highlighted by the use of a Cytomatrix block, which is typically employed to enhance sample adequacy when conventional smears provide insufficient material. Although Cytomatrix has been successfully used in other studies to improve cellular retention and allow more comprehensive cytological evaluation, the extensive fibroinflammatory process characteristic of Riedel’s thyroiditis resulted in a severely limited cellular yield [1,28]. As a consequence, the Cytomatrix cell block was inconclusive, offering no additional clarification beyond the misleading cytologic features observed on the smear. When correlated with the final histological diagnosis, the fibrotic material observed in the CytoMatrix block was compatible with the underlying fibrosclerotic process of Riedel’s thyroiditis. This finding underscores that, in profoundly fibrotic thyroid lesions, even advanced adjunctive cytologic techniques may be unable to retrieve adequate follicular elements, thereby perpetuating the risk of misclassification and highlighting the intrinsic limitations of preoperative cytologic assessment in Riedel’s thyroiditis [24,25].

Cross-sectional imaging such as contrast-enhanced CT may provide valuable information regarding the extent of local invasion and is recommended when aggressive thyroid malignancies such as anaplastic thyroid carcinoma are suspected. In the present case, CT imaging was not performed preoperatively, which represents a limitation of the diagnostic pathway. The patient had undergone endocrinological evaluation, including thyroid ultrasonography and FNAC, prior to referral to the surgical department and was subsequently admitted because of progressive compressive symptoms associated with the rapidly enlarging thyroid gland. Definitive diagnosis typically relies on histopathological examination. Additional tissue sampling with core-needle biopsy could also have provided further preoperative histologic information; however, this procedure was declined by the patient.

Intraoperatively, the thyroid was diffusely fibrotic and firm and densely adherent to surrounding muscles, closely mimicking an infiltrative carcinoma. To clarify the ambiguous intraoperative findings and help determine the appropriate extent of dissection, an intraoperative frozen-section examination was requested. Microscopy revealed dense fibrous and collagenous tissue with chronic inflammatory infiltrate containing eosinophils, without malignant cellular features—findings consistent with Riedel’s thyroiditis.

The frozen-section findings helped guide intraoperative management. Rather than proceeding with an extensive oncologic resection under the assumption of malignancy, the surgical team performed a near-total thyroidectomy, allowing removal of the majority of the fibrotic gland while preserving critical structures such as the recurrent laryngeal nerves and parathyroid glands. In this context, intraoperative pathological assessment assisted in tailoring the surgical approach while minimizing the risk of complications associated with overly aggressive resection.

Although radical thyroidectomy is generally avoided in Riedel’s thyroiditis because of the high risk of complications associated with diffuse fibrosis, surgery remains essential for both diagnosis and symptomatic relief. In our patient, postoperative recovery was favorable, with resolution of compressive symptoms over several months under continued medical therapy [29,30,31].

Different surgical strategies have been described. To relieve airway compression while minimizing complications, limited resections such as wedge isthmectomy or partial lobectomy are commonly preferred [13,22]. In the series by Zala et al. [32], 82% of 212 patients underwent surgery; 34% received total thyroidectomy and 48% other, more conservative procedures such as tracheostomy, isthmectomy, or hemithyroidectomy. These data highlight that the extent of surgery in Riedel’s thyroiditis often needs to be determined intraoperatively. In this context, intraoperative frozen-section examination can provide valuable information to help guide surgical decision-making, particularly when preoperative findings remain inconclusive.

In our case, another surgical team had initially proposed tracheostomy because of the extensive fibrosis associated with the progressive compressive symptoms. However, the procedure was ultimately performed by a surgical team with extensive thyroid surgery experience, allowing near-total thyroidectomy with preservation of vital structures. This outcome illustrates how intraoperative pathological assessment, combined with surgical expertise, may assist in tailoring the extent of surgery in complex cases of Riedel’s thyroiditis.

Minimizing residual thyroid tissue may help reduce the risk of recurrence and decrease the need for prolonged corticosteroid or tamoxifen therapy, which can lead to adverse effects such as thrombosis reported after long-term tamoxifen use [13,33].

Although preoperative investigations may raise suspicion for malignancy, definitive differentiation between Riedel’s thyroiditis and anaplastic carcinoma can remain challenging. Histopathologic confirmation therefore plays a crucial role in establishing the diagnosis. In many cases, core-needle biopsy may provide valuable preoperative histologic information; however, in the present case this procedure was declined by the patient, and definitive diagnosis was ultimately established following surgical excision. Even then, interpretation may occasionally be difficult, as rare atypical anaplastic carcinomas can mimic the fibrosing pattern seen in Riedel’s thyroiditis [33].

Both frozen-section and paraffin-embedded examinations in this case supported the diagnosis of Riedel’s thyroiditis. As demonstrated by several studies, intraoperative frozen-section examination can be useful in differentiating benign from malignant pathology [12,22,34] and may contribute to intraoperative decision-making when diagnostic uncertainty persists. In our case, the frozen-section findings helped guide the extent of surgery, allowing the surgical team to obtain diagnostic confirmation and achieve adequate decompression while avoiding an unnecessarily radical procedure.

Although not performed in this case, immunohistochemical analysis of the excised tissue can demonstrate IgG4-positive plasma cell infiltration, a hallmark of IgG4-related disease [26,35,36].

Glucocorticoids remain the first-line medical treatment for Riedel’s thyroiditis. Multiple reports describe symptomatic and radiologic improvement after steroid therapy, though others have shown limited benefit [37,38,39,40]. Early initiation appears to correlate with better outcomes [38,40,41]. Tamoxifen has also shown efficacy through inhibition of fibroblast proliferation via TGF-β stimulation [13]. In one study of four refractory cases, all patients demonstrated marked regression after tamoxifen therapy [42].

## 4. Conclusions

Riedel’s thyroiditis remains a rare and diagnostically challenging thyroid disorder that can closely mimic malignancy both clinically and cytologically. As demonstrated in this case, preoperative fine-needle aspiration cytology may be misleading and may raise suspicion of carcinoma. Intraoperative frozen-section examination may provide useful information when preoperative findings remain inconclusive and may help guide surgical decision-making. In our patient, the intraoperative findings supported a benign fibroinflammatory process consistent with Riedel’s thyroiditis and allowed the surgical team to limit the extent of resection, avoiding an unnecessarily aggressive procedure while achieving relief of compressive symptom minimizing the risk of disease persistence or recurrence.

This diagnostic challenge may also arise in other fibrosing thyroid conditions within the spectrum of IgG4-related thyroid disease, such as invasive variants of Hashimoto’s thyroiditis, in which preoperative cytologic assessment may be limited and the risk of overtreatment exists. This case further highlights the importance of multidisciplinary collaboration among surgeons, pathologists, and endocrinologists in establishing the diagnosis and planning appropriate management. When combined with appropriate medical therapy, surgery can contribute both to diagnostic clarification and to the relief of compressive symptoms, thereby improving patient outcomes.

## Figures and Tables

**Table 1:** Laboratory findings at admission.

Parameter	Result	Reference Range
Thyroid-stimulating hormone (TSH)	9.50 mIU/L	0.46–4.68 mIU/L
Free thyroxine (FT4)	24.2 pmol/L	10.0–28.2 pmol/L
Thyroglobulin antibodies (anti-TG)	1885 IU/mL	0–115 IU/mL
Thyroid peroxidase antibodies (anti-TPO)	>1000 IU/mL	0–34 IU/mL
White blood cells	10,090/µL	4000–9500/µL
Hemoglobin	10.5 g/dL	11.5–15 g/dL
Serum calcium	8.3 mg/dL	8.4–10.2 mg/dL
Fibrinogen	604 mg/dL	200–393 mg/dL
Serum IgG4	0.271 g/L	0.030–2.010 g/L

## Data Availability

All relevant data supporting the findings of this study are included within the article.

## Abbreviations

The following abbreviations are used in this manuscript:

FNAC	Fine-Needle Aspiration Cytology
TSH	Thyroid-Stimulating Hormone
FT4	Free Thyroxine
anti-TPO	Anti-Thyroid Peroxidase Antibodies
anti-TG	Anti-Thyroglobulin Antibodies
IgG4	Immunoglobulin G4
IgG4-RD	Immunoglobulin G4–Related Disease
B-mode	Brightness-Mode Ultrasound
SWE	Shear-Wave Elastography
2D-SWE	Two-Dimensional Shear-Wave Elastography
kPa	Kilopascal
RLN	Recurrent Laryngeal Nerve
LRLN	Left Recurrent Laryngeal Nerve
TGF-β	Transforming Growth Factor-Beta
